# Cathepsin K inhibition preserves compressive load in lumbar vertebrae of osteoporotic monkeys

**DOI:** 10.1016/j.bonr.2018.10.001

**Published:** 2018-10-18

**Authors:** Isabel D. Colón-Bernal, Le T. Duong, Brenda Pennypacker, James Henderson, Kenneth M. Kozloff, Mark M. Banaszak Holl

**Affiliations:** a Chemistry Department, University of Michigan, Ann Arbor, MI, USA; b Bone Biology Group, Merck & Co., Inc., West Point, PA, USA; c Center for Statistical Consultation and Research (CSCAR), University of Michigan, Ann Arbor, MI, USA; d Department of Orthopaedic Surgery, University of Michigan, Ann Arbor, MI, USA; e Biomedical Engineering, University of Michigan, Ann Arbor, MI, USA; f Department of Chemical Engineering, Monash University, Melbourne, Australia

**Keywords:** Osteoporosis, Cathepsin-k, Alendronate, Micro computed tomography

## Abstract

Anti-resorptive drugs treat bone loss by blocking osteoclast activity through a variety of mechanisms of action. Once significant bone loss has occurred, the ability to restore biomechanical function may differ based on the drug chosen. To assess this question, bisphosphonate (alendronate, ALN) and cathepsin K inhibitor (MK-0674, CatKi) were employed in treatment mode to compare the relative changes to cancellous bone microstructure and mechanical properties in ovariectomized (OVX) cynomolgus monkeys. Lumbar vertebrae (LV) bone mineral density (BMD) values taken two years post-surgery prior to drug treatment show a 10–15% decrease (p < 0.05) for all OVX animals. OVX animals were then treated with vehicle (VEH), ALN (0.03 mg/kg weekly), or CatKi MK-0674 (0.6 or 2.5 mg/kg daily, CatKi-L and H respectively) for two years and compared to a control Sham surgery group. Ex-vivo microcomputed tomography (μCT) of LV2 and compression testing of LV4–6 were used to measure cancellous bone microstructure and changes in bone mechanics, respectively. After two years of treatment, ALN-treated animals showed no significant difference in μCT or biomechanical parameters when compared to Veh. However, treatment with CatKi-H resulted in a 30% increase in yield and peak loads, and apparent peak and yield stress as compared to Veh (p < 0.05) and gave average mechanical values greater than the Sham sample. Treatment with CatKi-L exhibited a similar trend of increase to CatKi-H (p < 0.08). Intriguingly, these changes were realized despite no significant differences in mean values of trabecular bone morphologic parameters. Together these data suggest matrix-level changes in bone composition that are unique to the CatK inhibition mechanism, resulting in the preservation of bone compressive load with treatment.

## 1 Introduction

Osteoporosis is characterized by a net bone loss due to an increase in bone resorption (Chen and Sambrook, 2011). Once significant bone loss has occurred, restoring biomechanical function is a primary challenge for interventional therapies (Campbell et al., 2008; Duong et al., 2016a). Antiresorptive treatments are widely used in the clinic to prevent bone loss through a variety of mechanisms of action (Chen and Sambrook, 2011; Duong et al., 2016a). The bisphosphonate Alendronate (ALN) adsorbs to the mineral in bone, and interferes with a key enzyme in the mevalonate pathway when taken up by the cell during bone resorption, leading to decreased osteoclast activity (Fisher et al., 1999; Russell et al., 2008).

Despite the effectiveness of ALN, serious but rare associated adverse effects can occur such as atypical fractures, atrial fibrillation, and osteonecrosis of the jaw (Jha et al., 2015; Jan et al., 2012; Drake et al., 2008; Kim et al., 2016). While these associated adverse effects are extremely rare and occur more commonly when bisphosphonates are used to treat cancer instead of osteoporosis, statements released by the Food and Drug Administration have raised concern regarding bisphosphonate use (Kim et al., 2016). This has led to a crisis in the treatment of osteoporosis due to high-risk patients denying treatment despite a beneficial clinical outlook (Khosla et al., 2017; Black and Rosen, 2016). Therefore, new drugs, as well as a better understanding of antiresorptive mechanisms, are desired.

Recently, attention has been directed toward interventions that induce partial osteoclast inhibitory effects as promising alternatives to complete osteoclast inhibition. The inhibition of Cathepsin K (CatK), a lysosomal cysteine protease found in osteoclasts that is responsible for degrading Type I collagen (Duong et al., 2015), is considered one of those promising alternatives. Cathepsin K-inhibition (CatKi) does not interrupt osteoclast to osteoblast communication, allowing bone formation to occur even though resorption is decreased through inhibition of protease activity (Bonnet et al., 2017; Sims and Ng, 2014; Bandeira and Bilezikian, 2017). The effectiveness of pharmacological cathepsin K inhibition as a treatment for bone diseases such as osteoporosis and osteoarthritis has been demonstrated in the literature (Sims and Ng, 2014; Bandeira and Bilezikian, 2017; Zhuo et al., 2014; Masarachia et al., 2012; Williams et al., 2013; Paschalis et al., 2017).

Previous studies of the effectiveness of CatKi in treating ovariectomized (OVX) skeletally mature rhesus monkeys focused on prevention-model studies where the drug is given immediately after the OVX surgery. Masarachia et al. noted an increase in lumbar vertebrae (LV) mineral density (BMD) upon treatment of skeletally mature rhesus monkeys with either 6 or 30 mg/kg Odanacatib (ODN) (Masarachia et al., 2012). A corresponding increase in the values of bone peak load and area under the curve were observed in mechanical testing, demonstrating functional significance to treatment-induced bone mass gains (Masarachia et al., 2012). Jerome et al. also noted a partial prevention of bone loss for monkeys treated with 3 or 10 mg/kg Balicatib (Jerome et al., 2012). In a comparative study with alendronate (ALN), Williams et al. noted that 2 mg/kg dose of ODN given to monkeys resulted in improvement in lumbar spine areal BMD (aBMD) and trabecular volumetric BMD (vBMD); however, higher doses of 4 and 8 mg/kg did not further improve these properties (Williams et al., 2013). Treatment effects were not limited to structural gains, but also induced a shift in the calcium distribution to higher average values, as assessed using quantitative backscattered electron imaging (qBEI) (Fratzl-Zelman et al., 2013).

Importantly, none of these prior studies examined the effect of cathepsin K inhibition following a period of bone mass depletion, reflecting the clinical state of osteoporosis. In the present study, alendronate (ALN), and a cathepsin K inhibitor (CatKi, MK-0674) were employed in rescue mode to explore the impact of varying biological mechanisms of action on changes to trabecular bone microstructure and biomechanical properties after two years of estrogen depletion in OVX cynomolgus monkeys. This animal model is especially relevant for the study of osteoporosis (Smith et al., 2009). To our knowledge, it is the first time that cathepsin K inhibition has been applied in a non-human primate study of onset osteoporosis treatment. The findings from our present study demonstrate that treatment following a period of trabecular bone depletion induces changes in biomechanical indices that are not reflected by similar improvements in structural indices of trabecular architecture. Together, this data suggests that cathepsin K inhibition may induce matrix-level changes that partially correct for deficiencies in biomechanical predictors of fracture.

## 2 Methods

### 2.1 Cynomolgus monkeys, diets, surgery and dosing with CatKI

The in-life portion of this study was conducted at Charles River Laboratories (CRL, Montreal, Canada) with the approval of IACUCs from both CRL and Merck & Co., Inc., Kenilworth, NJ, USA. This study protocol was conducted essentially based on the study protocol of the Odanacatib Research Rhesus Investigational Study (ORRIS) (Williams et al., 2013). Previously in the ORRIS study all drug dosing, including the CatKI odanacatib (ODN) were administered in Rhesus monkeys in prevention mode (right after OVX surgery). In contrast, in this study, all drug dosing including the CatKI MK-0674 (Isabel et al., 2010) were dosed on Cynomolgus monkeys in treatment mode (initiated at 2 years post-OVX). Female Cynomolgus monkeys (10–27 years of age) were subjected to ovariectomy (OVX) or sham surgery. All monkeys were subsequently randomized into groups by weight and lumbar spine (LV1–4) bone mineral density (LsBMD).

**Scheme 1:**
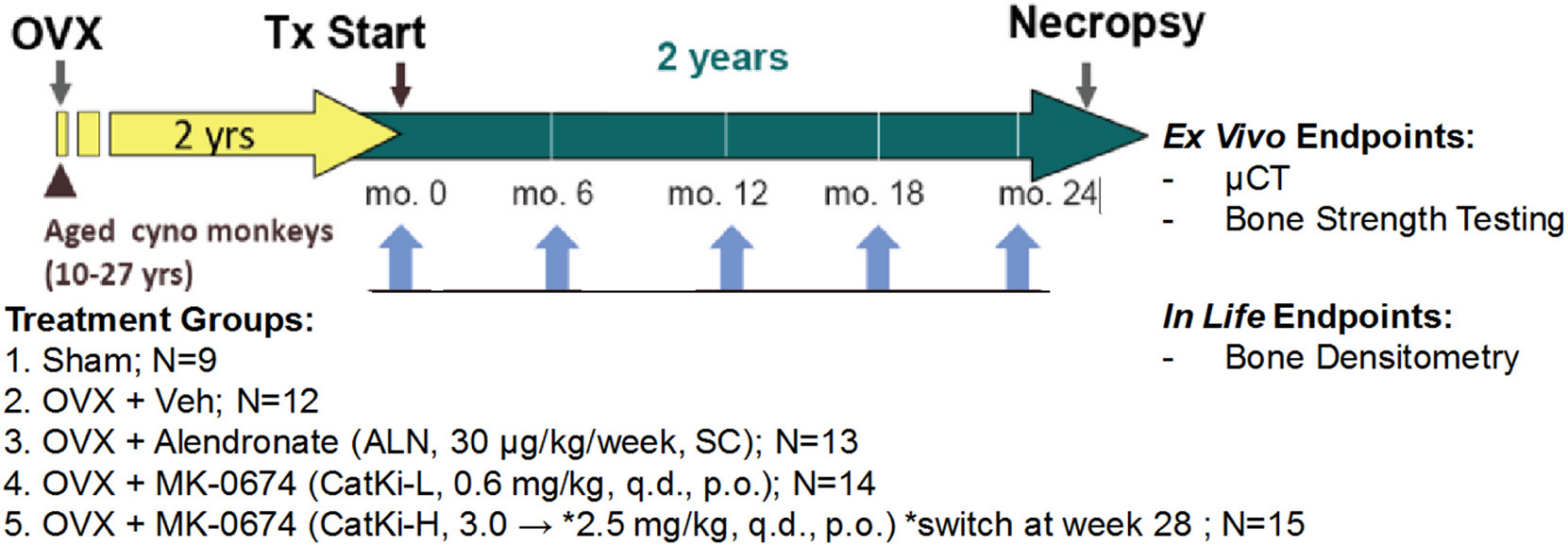
Treatment animal study timeline. Cynomolgus monkeys were treated with CatKi, and ALN for 24 months in rescue mode two years post-surgery. Intact (Sham), and a disease (OVX + Veh) groups were included. *In*-*life* bone densitometry was evaluated every six months as shown above with the blue arrows. Ex vivo μCT, and bone compression testing were performed on LVs 4 and 5. (For interpretation of the references to color in this scheme legend, the reader is referred to the web version of this article.)

Sham animals (n = 16) were dosed daily with vehicle (hydroxypropyl methyl cellulose acetate succinate (HPMC-AS) polymer), p.o. OVX animals were randomized into 4 groups, vehicle (n = 16), MK-0674 at 0.6 mg/kg p.o., q.d., (AUC = 10.9 ± 4.5; n = 16), MK-674 at 2.5 mg/kg p.o., q.d (AUC = 69.1 ± 28.7; n = 16), or Alendronate (ALN) at 30 mg/kg/wk. s.c. once-weekly (n = 16). Drug administration was continued for 24-months. Prior to necropsy, bone mineral density of the lumbar spine was monitored with dual energy X-ray absorptiometry (DXA) and peripheral Quantitative Computed Tomography (pQCT) at baseline (before randomization and before dosing started), and approximately at 3, 6, 9, 12, 16, and 24-months after drug administration initiated. For a visual summary see Scheme 1.

### 2.2 Dual energy X-ray absorptiometry (DXA) and Computed Tomography (CT) imaging

The animals were anesthetized by ketamine and maintained under isoflurane as for OVX. In vivo areal BMD, bone mineral content (BMC) and bone area of the lumbar vertebrae LV1 to LV4 were measured by DXA using a Hologic QDR Discovery A bone densitometer (Hologic, Inc., Waltham, MA). Ex vivo Peripheral QCT scans were acquired as single slice using a XCT Research SA or SA + bone scanner with software version 5.50D (Stratec medizintechnik; Pforzheim, Germany) (Masarachia et al., 2012; Pennypacker et al., 2014).

Ex vivo volumetric measures of total and trabecular BMD and BMC of vertebral bodies (LV4 and LV5) were obtained from a 0.8 mm-thick slice taken from the middle of each vertebral body excised for biomechanical testing (Duong et al., 2016a). Another vertebral site (LV2) was further subjected to μCT scanning at an isotropic 20 μm resolution using a Scanco μCT-40 scanner (Scanco Medical AG, Brütisellen, Switzerland) as previously described (Cabal et al., 2017).

μCT scans were analyzed using MicroView. Original scans had to be cropped using MicroView Parallax (Ilderton, ON) due to file size limitations, and a median filter was applied to reduce noise. A region of interest (ROI) was defined from superior growth plate to inferior growth plate for each sample. Trabecular bone was contoured every 10 slices, and a 3D ROI was defined within the contoured area. The ROI was analyzed to obtain trabecular morphologic parameters, see Table 1.

**Table 1:** Bone densitometry, microstructure, and bone compression results and statistical analysis.

Parameter	Sham	OVX			
		Veh	CatKI L	CatKI H	ALN
	N = 9	N = 12	N = 14	N = 15	N = 13
In-life pre-treatment					
Bone mineral density (g/cm^2^)	0.781 (0.068)⁎⁎⁎	0.695 (0.072)	0.698 (0.091)	0.662 (0.090)	0.674 (0.066)

Post-treatment					
Trabecular number (1/mm)	2.30 (0.23)	2.15 (0.24)	2.24 (0.18)	2.15 (0.35)	2.18 (0.23)
Trabecular thickness (mm)	0.185 (0.055)	0.168 (0.037)	0.194 (0.055)	0.175 (0.027)	0.177 (0.036)
Connectivity density (1/mm^3^)	8.6 (4.2)	7.9 (4.2)	6.0 (3.9)	6.8 (2.9)	7.8 (1.8)
Vertebral height (mm)	7.43 (0.11)	7.35 (0.22)	7.35 (0.11)	7.39 (0.12)	7.355 (0.14)
Peak load (N)	3097 (550)	2620 (580)	3460 (1100)⁎	3507 (860)⁎⁎	3053 (490)
Apparent peak stress (MPa)	27.0 (3.6)	24.3 (4.0)	32.9 (11)⁎⁎	32.5 (7.5)⁎	26.6 (4.8)
Yield load (N)	2660 (450)	2280 (490)	2980 (940)⁎	2974 (690)⁎	2678 (400)
Yield stress (MPa)	23.1 (2.8)	21.2 (3.3)	28.3 (9.3)⁎⁎	27.5 (5.6)⁎	23.3 (3.6)
Stiffness (N/mm)	18,750 (3800)	16,630 (4300)	20,280 (4300)	20,530 (4100)	19,480 (2600)
Modulus (MPa)	1214 (220)	1122 (210)	1420 (370)⁎	1400 (250)	1250 (190)
Area under the curve (N/mm)	907 (330)	673 (240)	1224 (960)	1214 (780)	628 (190)
Toughness (F)	1.08 (0.41)	0.85 (0.28)	1.6 (1.4)	1.5 (1.0)	0.76 (0.28)

Data are presented as means ± (SD), and rounded to represent significant figures based on SD; BMD presented was collected after two years of estrogen depletion and prior to treatment.

⁎ p < 0.1 vs Veh.

⁎⁎ p < 0.05 vs Veh.

⁎⁎⁎ p < 0.5 vs Veh and other treatments.

### 2.3 Biomechanical testing

Bones were thawed overnight in a refrigerator at 4 °C prior to μCT and bone strength testing. Vertebrae were trimmed, removing dorsal elements, spinal processes, and the cartilaginous endplates with a diamond saw leaving only the cartilage-free vertebral body with plano-parallel ends. Biomechanical testing of the vertebral bodies (LV4 and LV5) was performed using the 858 Mini Bionix Servohydraulic Test System, Model 242.03 (Duong et al., 2016a). All data were collected in an Excel Spreadsheet which was used to manually derive the required biomechanics parameters. The height of the vertebral body test specimen was measured using digital calipers before compression testing with a load cell of 15 kN and a loading rate of 20 mm/min. Peak load was recorded as the maximum of the load–displacement curve and stiffness were defined as the slope of the linear portion (Turner, 2002). Work, the energy required to break the bone, was calculated as the area under the curve to peak load for compression tests. The apparent yield and peak stress were calculated using the yield load and peak load divided by the cross-sectional area respectively. The area was determined by peripheral quantitative tomography (data not presented here). Modulus was calculated using stiffness, thickness and area, see Eq. (1) (Duong et al., 2016a; Turner, 2002). Toughness was calculated using work-to-failure, thickness and area, see Eq. (2) (Duong et al., 2016a).

(1) ModulusMPa=StiffnessNmm∗thickness of specimenmmAreamm2

(2) ToughnessMPa=Area Under the CurveNmm(Areamm2∗thickness of specimenmm

### 2.4 Statistical analysis

Microstructure and biomechanical results are reported as means ± SD, as seen in Table 1. Statistical analysis was performed using R. A one-way analysis of variance (ANOVA) was used to determine treatment group differences for each specific variable. Tukey's Test was used to detect differences in means between specific treatment groups.

## 3 Results

### 3.1 OVX induces trabecular bone loss over a 2-year depletion period

**Fig. 1:**
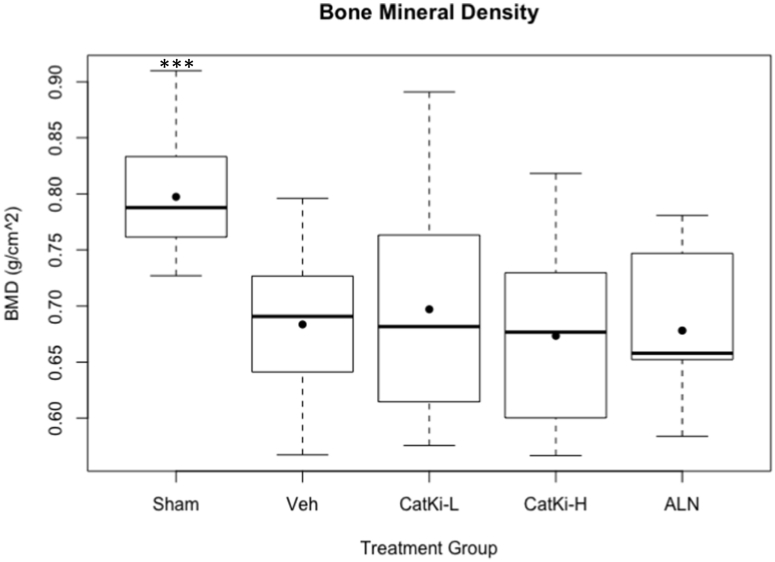
*In Life* DXA assessment of Bone Mineral Density 2 years after surgery, before start of treatment, shows 10–15% reduction in BMD at the lumbar vertebrae for all OVX groups when compared to Sham group. ***p < 0.05 vs. Veh, CatKi-L and -H, ALN. Box and Whisker plots showing mean (filled dot), median (line), 25% and 75% percentile (lower and upper quartile, respectively), maximum and minimum values (lower and upper whiskers, respectively), and outliers (circle).

Two years after initial surgery, but prior to drug treatment, all experimental groups demonstrated similar loss (10–15%) of bone mineral density (BMD) at LV when compared to Sham-operated controls (Table 1 and Fig. 1) with no difference between individual groups, indicating a well-balanced study allocation. Bone loss was consistent with previous work demonstrating an 11% decrease in BMD for OVX animals vs. sham controls over 21 months (Masarachia et al., 2012).

### 3.2 2-Year treatment following trabecular depletion shows no effect on trabecular microarchitecture

Ex vivo μCT scans performed on LV2 after 2 years of treatment demonstrated no significant differences in trabecular bone architectural parameters between Sham animals and any treatment group for bone volume fraction (BVF), trabecular number (Tb.N.), trabecular thickness (Tb.Th.), or connectivity density (Table 1).

### 3.3 Cathepsin K inhibition preserves biomechanical indices

**Fig. 2:**
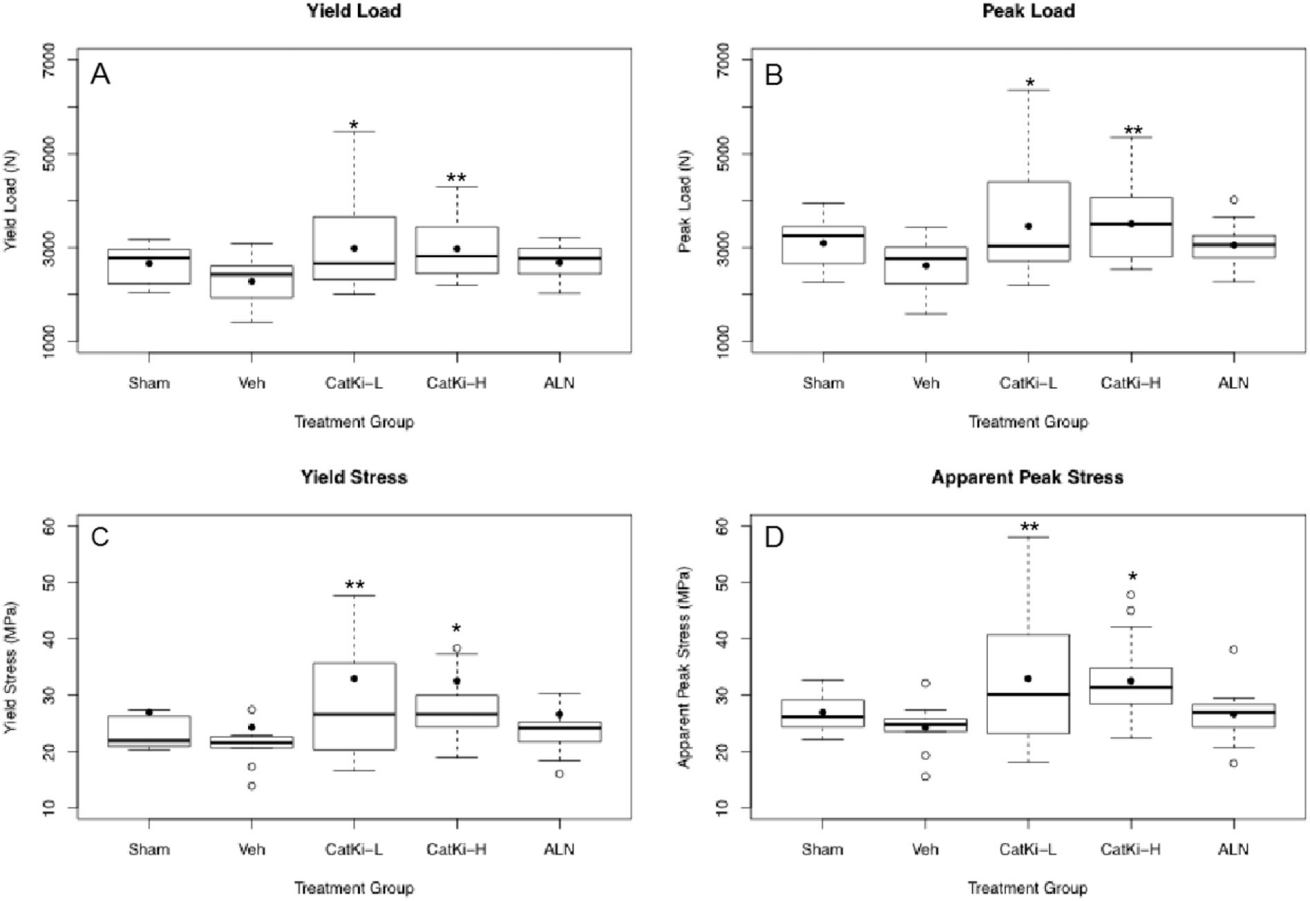
Biomechanical testing shows significant (~30%) increase in yield load (panel A), peak load (panel B), yield stress (panel C), and apparent peak stress (panel D) after 2 years of CatKi treatment vs. Veh animals. No significant difference was observed in ALN animals vs. Veh. *p < 0.1 vs Veh; **p < 0.05 vs. Veh. Box and Whisker plots showing mean (filled dot), median (line), 25% and 75% percentile (lower and upper quartile, respectively), maximum and minimum values (lower and upper whiskers, respectively), and outliers (circle).

Compression testing was performed on LV4–5. Values presented in Table 1 are an average of LV4 and LV5. OVX induces modest decreases in yield and peak loads vs. sham controls as observed in Table 1. While ALN-treated vertebrae showed no differences vs. Sham or OVX-Veh, CatKi-L and CatKi-H increased yield and peak loads, and apparent yield and peak stress to levels significantly greater than OVX-Veh (Fig. 2). All intervention biomechanical parameters were statistically indistinguishable from Sham controls (Fig. 2), suggesting a preservation of effects of OVX itself.

## 4 Discussion

Treatment with CatKi has been able to preserve biomechanical properties in NHP osteoporosis/bone loss prevention models through a unique mechanism of action (Duong et al., 2016a). In the present study, biomechanical properties of lumbar vertebrae were increased with CatKi treatment administered to NHP in rescue mode. Specifically, yield and peak loads as well as apparent yield and peak stress were significantly increased between CatKi treated groups and OVX-Veh treatments. However, no detectable changes in bone microstructure by μCT analysis were observed. These improvements in biomechanical properties are similar to results observed in prevention mode studies done on rhesus monkeys.

Masarachia et al. assessed the long-term (21-months) effects of the ODN on newly OVX rhesus monkey (Masarachia et al., 2012). In that study, the effects of ODN on reduction of trabecular bone turnover, prevention of estrogen deficiency–induced bone loss, and a significant increase in lumbar spine BMD were observed (Masarachia et al., 2012). This increase highly correlated to higher bone peak loads indicating a preservation of biomechanical properties after treatment (Masarachia et al., 2012). These results were reproduced in radius and tibia (Jayakar et al., 2012), hip (Cusick et al., 2012), and femurs (Pennypacker et al., 2014) demonstrating that CatKi preserves biomechanical properties of bone while inhibiting resorption. Our results are consistent with Masarachia et al.'s results of biomechanical properties preservation after treatment with CatKi in lumbar vertebrae of rhesus monkeys. Increase in BMD, inhibition of trabecular bone turnover, while stimulation of periosteal bone formation in femurs was observed in cynomolgus monkeys treated with Balicatib, another CatK inhibitor (Jerome et al., 2012).

In the ODN study in rhesus monkeys, Williams et al. demonstrated ODN dosing at approximated clinical exposure for 21 months in prevention mode increased lumbar spine aBMD (areal BMD), and trabecular vBMD (volumetric BMD) with comparable efficacy to ALN (Williams et al., 2013). Higher dose of ODN showed no additional efficacy. In that same study, Duong et al. evaluated the long-term effects of ODN versus ALN in the prevention of bone microstructure deterioration (Duong et al., 2016a). At the end of the study, no significant differences in BMD (both cortical and trabecular), trabecular microstructure, and biomechanical properties including peak load and stiffness were observed in lumbar vertebrae from the treatment groups, unlike this study (Duong et al., 2016a). However, our results of no detectable trabecular microstructure change or stiffness is consistent with Duong et al.'s study.

In the present study, we compared CatKi to ALN dosed in intervention mode. CatKi-L and CatKi-H treatments differentiated from ALN by increased yield and peak loads over Veh. ALN treatment was no different than Sham and Veh groups. The changes in mechanical properties, without concomitant changes in bone microstructure, suggest that important changes in bone material composition may contribute to the observed mechanical difference, further supported by the observed increase in calculated apparent yield and peak stress. Compositional changes in bone have been associated with osteoporosis, and heterogeneity of these measurements is also emerging as an important contributor to bone mechanical properties, and fragility fracture risk (Lloyd et al., 2015; Boskey et al., 2016; Donnelly et al., 2012; Tai et al., 2007; Wang et al., 2016; Demirtas et al., 2016; Gourion-Arsiquaud et al., 2010; Vennin et al., 2017; Yao et al., 2011). Nano- to microscale bone heterogeneity likely plays a crucial role in understanding bone mechanical properties and their role in fracture-risk (Lloyd et al., 2015; Boskey et al., 2016; Donnelly et al., 2012; Tai et al., 2007; Wang et al., 2016; Demirtas et al., 2016; Gourion-Arsiquaud et al., 2010; Vennin et al., 2017; Yao et al., 2011). The present findings suggest that inhibition of Cathepsin K may preserve, or reverse compositional changes and improve vertebral biomechanics with treatment.

Studies have shown how reductions in compositional heterogeneity can affect tissue-level toughening mechanisms (Donnelly et al., 2012; Gourion-Arsiquaud et al., 2010). In addition, lack of heterogeneity in hardness and modulus has been observed to worsen bone quality at the nano-scale in cortical and trabecular bone (Vennin et al., 2017). Taking this into consideration, inelasticity variation was also explored experimentally and computationally (Tai et al., 2007; Yao et al., 2011). These studies found that inelasticity plays a dominant role in energy dissipation in bone (Tai et al., 2007; Yao et al., 2011). Our study suggests potential for multiple regulators of bone mechanical function beyond bone quantity and structural organization alone.

This study has several limitations. First, the ovariectomy may have not produced a strong biomechanical phenotype because of genetic variability in our samples. Our animal model was designed to mimic the treatment of osteoporosis in the clinic. Therefore, the subjects were not genetically identical contrary to most rodent models. However, that we did not observe a difference between OVX and Sham controls was consistent with previous prevention studies in rhesus monkeys (Duong et al., 2016a) and rabbits (Duong et al., 2016b) that were treated with ODN. Second, an intermittent parathyroid hormone (iPTH) control group would have added value to this study as a positive control, as well as providing a deeper understanding of how CatKi compares to an anabolic drug when administered after a significant period of estrogen depletion. In an analogous study, iPTH was administered to Rhesus monkeys for 16 months following 9 months of estrogen depletion (Fox et al., 2007). Similar to our present findings, Fox et al. reported that treatment with iPTH for 16 months resulted in a dose-dependent increase in lumbar spine yield load and stiffness compared to Veh animals to values that were statistically no different than sham controls (Fox et al., 2007). The magnitude of response appears greater with iPTH, and this may reflect the mechanism of action of drug, along with the difference in magnitude of bone loss between the two studies in the depletion period (Fox et al. 5% BMD depletion, present study 10–15% BMD depletion). Third, cortical bone was not evaluated in the present study. Recently, it was proposed that CatK degrades periostin thereby modulating Wnt-b-catenin signaling and generating higher osteoblast activity in cortical versus trabecular bone (Bonnet et al., 2017). This mechanism suggests that cortical bone volume can increase in response to CatK inhibition and several prevention mode studies have observed that CatKi promotes new periosteal surface bone formation in tibia (Jayakar et al., 2012), hip (Cusick et al., 2012), and femurs (Pennypacker et al., 2014). The potential role of such effects for vertebrae is an important area for future study.

## 5 Conclusion

In conclusion, increases in yield and peak load were observed for CatKi treatment when compared to OVX-Veh despite no significant differences in trabecular architectural parameters as measured by μCT. Treatment with CatKi resulted in a 30% increase in yield and peak loads, and apparent yield and peak stress when compared to Veh. This preservation of biomechanical properties appears to be unique to cathepsin K inhibition, as compared to alendronate. This is the first time a preservation in mechanical properties has been observed for an antiresorptive osteoporosis drug administered in treatment mode. Improvement of biomechanical properties is one of the current challenges in the field of osteoporosis.

## Transparency document

Transparency document. Image 1
